# Curcumin Attenuates Adipogenesis by Inducing Preadipocyte Apoptosis and Inhibiting Adipocyte Differentiation

**DOI:** 10.3390/nu11102307

**Published:** 2019-09-28

**Authors:** Liang-Yi Wu, Chien-Wei Chen, Luen-Kui Chen, Hsiang-Yun Chou, Chih-Ling Chang, Chi-Chang Juan

**Affiliations:** 1 Department of Bioscience Technology, College of Science, Chung-Yuan Christian University, Chung Li 32023, Taiwan; lywu@cycu.edu.tw; 2 Department of Physiology, School of Medicine, National Yang-Ming University, Taipei 11221, Taiwan; gogozipper130@gmail.com (C.-W.C.); luenkui0506@gmail.com (L.-K.C.); convey07@hotmail.com (H.-Y.C.); forrest70312@gmail.com (C.-L.C.); 3 College of Human Development and Health, National Taipei University of Nursing and Health Sciences, Taipei 11219, Taiwan; 4 Department of Medical Research and Education, Taipei Veterans General Hospital, Taipei 11217, Taiwan

**Keywords:** curcumin, adipocytes, adipogenesis, apoptosis, mitotic clonal expansion, differentiation

## Abstract

Patients with metabolic syndrome are at an increased risk of developing type 2 diabetes and cardiovascular diseases. The principal risk factor for development of metabolic syndrome is obesity, defined as a state of pathological hyperplasia or/and hypertrophy of adipose tissue. The number of mature adipocytes is determined by adipocyte differentiation from preadipocytes. The purpose of the present study is to investigate the effects of curcumin on adipogenesis and the underlying mechanism. To examine cell toxicity of curcumin, 3T3-L1 preadipocytes were treated with 0–50 µM curcumin for 24, 48, or 72 h, then cell viability was measured using the MTT (3-(4,5-dimethylthiazol-2-yl)-2,5-diphenyltetrazolium bromide) assay. The effect of curcumin on the cell cycle was determined by flow cytometry. Curcumin-induced cell apoptosis was determined by the TUNEL assay and curcumin-induced caspase activation was measured by immunoblotting. The effect of curcumin on adipocyte differentiation was determined by measuring mitotic clonal expansion (MCE), expression of adipogenic transcription factors, and lipid accumulation. Results showed the viability of preadipocytes was significantly decreased by treatment with 30 µM curcumin, a concentration that caused apoptosis in preadipocytes, as assessed by the TUNEL assay, and caused activation of caspases 8, 9, and 3. A non-cytotoxic dose of curcumin (15 µM) inhibited MCE, downregulated the expression of PPARγ and C/EBPα, prevented differentiation medium-induced β-catenin downregulation, and decreased the lipid accumulation in 3T3-L1 adipocytes. In conclusion, our data show that curcumin can induce preadipocyte apoptosis and inhibit adipocyte differentiation, leading to suppression of adipogenesis.

## 1. Introduction

Increased visceral fat mass is associated with the development of metabolic disorders, including insulin resistance, dyslipidemia, hypertension, atherosclerosis, and inflammation [1]. Obesity is considered as the main cause of metabolic disorders-related diseases, such as cardiovascular disease, hypertension, hyperlipidemia, type 2 diabetes, and cancer [2]. Thus, the prevention of abnormal adipogenesis could be an efficient strategy for preventing the development of metabolic disorder-related diseases [3,4]. 

Curcumin, a yellow phenolic component of turmeric, is derived from the rhizome of *Curcuma longa L*. and has antioxidant [5], anti-inflammation [6,7], and anti-cancer properties [8,9]. It also has several ameliorating effects on metabolic disorders. For example, dietary supplementation with curcumin significantly reduces obesity, inflammation, and diabetes in obese animal models [10,11,12]. Curcumin also inhibits TNFα-stimulated inflammatory cytokine expression in adipocytes [10] and suppresses preadipocyte differentiation in vitro [13]. Supplementation with curcumin significantly reduces the high-fat diet-induced increase in body weight gain and adiposity in mice [13,14]. A similar inhibition of differentiation of 3T3-L1 adipocytes was reported by Lee et al. [15], who also found that curcumin can induce apoptosis of MCF-7 breast cancer cells; however, the apoptotic effect of curcumin on preadipocytes was not investigated. In addition, although curcumin is known to have an anti-obesity effect [10,11,12,13,14,15], little is known about the underlying mechanisms of inhibition of adipogenesis.

The adipogenic process is divided into different stages of growth arrest, mitotic clonal expansion (MCE), and differentiation. Retinoblastoma protein (Rb), a tumor suppressor protein, plays an important role in the initial step of adipogenesis. Adipogenic hormones, such as isobutylmethylxanthine (IBMX), dexamethasone, and insulin, induce Rb phosphorylation through the cyclin-dependent kinase pathway, resulting in dissociation of the Rb/E2F complex and allowing E2F to promote cell-cycle progression to the S phase [16,17,18]. The next step in adipogenesis is the re-entry of growth-arrested preadipocytes into the cell cycle and the completion of several rounds of MCE. Several groups have reported that MCE is necessary for subsequent preadipocyte differentiation [19,20,21,22,23]. 

Adipocyte differentiation is a complex process that is mainly controlled by two families of transcription factors, CCAAT enhancer binding proteins (C/EBPs) and peroxisome proliferator-activated receptors (PPARs) [24,25]. Differentiation of preadipocytes is characterized by marked changes in the pattern of gene expression that are caused by the sequential induction of these transcription factors. Preadipocytes exposed to differentiation inducers show an early and transient increase in expression of C/EBPβ and C/EBPδ [26,27] which, in turn, contributes to cell proliferation and to the subsequent increase in expression of C/EBPα and PPARγ [28,29]. These last two proteins are thought to act synergistically in the transcriptional activation of a variety of adipocyte-specific genes, with each activating the expression of the other [30,31].

The other regulatory pathway of adipogenesis is the Wnt signaling pathway, which maintains preadipocytes in an undifferentiated state by inhibiting expression of the adipogenic transcription factors C/EBPα and PPARγ [32,33]. In 3T3-L1 adipocytes, curcumin is reported to activate phosphorylation of AMP-activated protein kinase (AMPK), thus downregulating the PPARγ expression, and to inhibit adipogenesis [15]. It also inhibits adipogenesis in 3T3-L1 adipocytes by restoring nuclear translocation of the Wnt signaling component β-catenin to a similar level compared to that in undifferentiated preadipocytes [34]. 

As aforementioned, the nutrient constituents of curcumin make it a potential candidate for the therapy of obesity. However, this possibility and underlying mechanisms remain to be proven largely. Hence, this study aims to describe the role of curcumin in the regulation of adipocyte generation and sought to elucidate the molecular mechanisms of the actions responsible.

## 2. Methods and Materials

### 2.1. Experimental Design

To explore dose and time effects of curcumin on the viability of 3T3-L1 preadipocytes, the cells were pretreated for 1 h with different concentration of curcumin (0, 10, 15, 30, 50 μM), then incubated with differentiation medium (see below) in the continued presence of the same concentration of curcumin for different times (0, 24, 48, 72 h), when cell viability was determined using the MTT assay. To further clarify the effect of curcumin on apoptosis, 3T3-L1 preadipocytes were pretreated for 1 h with 30 μM curcumin, then incubated for 24 h with differentiation medium in the continued presence of 30 μM curcumin, then apoptosis was evaluated using the TUNNEL assay. To gain an insight into the apoptosis signaling pathway involved, expression of caspase proteins was examined by immunoblotting. 

To explore the effect of curcumin on adipocyte differentiation, 3T3-L1 preadipocytes were pretreated for 1 h with different concentrations of curcumin (0, 5, 10, 15, 20 μM), then differentiation was induced by incubation for 10 days in the continued presence of the same concentration of curcumin. During the differentiation processes, MCE was measured by flow cytometry, phosphorylation or expression of adipogenic proteins (pRb, cyclin D_1_, C/EBPβ, p27, PPARγ, C/EBPα, and β-catenin) was measured by immunoblotting, and the efficiency of adipocyte differentiation was determined by measuring intracellular triglyceride (TG) content and by Oil red O staining. 

To test whether the PPARγ ligand rosiglitazone reversed the inhibitory effect of curcumin on differentiation, 3T3-L1 preadipocytes were incubated for 1 h with or without 0.5 µM rosiglitazone, then were incubated with or without 15 μM curcumin in the continued presence or absence of rosiglitazone for 3 days in differentiation medium, 3 days in complete medium containing 1.7 μM insulin and 3 days in complete medium. During the differentiation processes, MCE and the efficiency of adipocyte differentiation were measured.

### 2.2. Cell Culture

3T3-L1 preadipocytes (American Type Culture Collection, Rockville, MD) were seeded onto 60-mm dishes or 12-well plates (Falcon^®^, Becton Dickinson, NJ, USA) and grown and maintained in complete medium [Dulbecco’s modified Eagle’s (DME) high glucose medium containing 100 units/mL of penicillin and 100 μg/mL of streptomycin (all from Gibco BRL, Gaithersburg, MD, USA), and 10% fetal bovine serum (FBS) (Biological Industries, Kibbutz Beit Ha’Emek, Israel)] in 10% CO_2_. The cells were grown to 3 days post-confluency, then induced to differentiate by incubation for 3 days in differentiation medium, i.e., complete medium containing 0.5 mM IBMX, 0.5 μM dexamethasone, and 1.7 μM insulin (all from Sigma, St. Louis, MO, USA), 3 days in complete medium containing 1.7 μM insulin and 3 days in complete medium. The medium was then changed every 3 days until the cells were fully differentiated. Typically, by day 10, >95% of the preadipocytes had differentiated into adipocytes as determined by staining for lipid accumulation using Oil Red O. This study protocol was used in all experiments. 

### 2.3. Mitotic Clonal Expansion Assayed by Cell Counting or by the MTT Assay

Using direct cell counting, cell numbers were quantified after trypsinization using a hemocytometer. Using the MTT assay, cells were seeded onto 96-well plates at approximately 1 × 10^3^ cells/well in 100 μL of DMEM containing 0.5% FBS. After incubation with curcumin, 15 μL of 3-(4,5-dimethylthiazol-2-yl)-2,5-diphenyltetrazolium bromide (MTT; USB, Amersham Life sciences, Cleveland, OH, USA) was added (final concentration 0.5 mg/mL) for 4 h, then 100 μL of DMSO was added to dissolve the formazan crystals formed and the optical density was measured on an ELISA plate reader using test and reference wavelengths of 570 and 630 nm, respectively. 

### 2.4. Propidium Iodide Staining and Flow Cytometry Analysis

Treated cells were trypsinized, washed with phosphate-buffered saline (PBS), and fixed overnight in 75% ethanol. The cells were washed twice with PBS and the cell pellet resuspended in cold PBS, then the cells were incubated at room temperature for 5–10 minutes with PBS containing 25 μg/mL of propidium iodide (PI), 0.5 mg/mL of RNase A, and 0.1% Triton X-100. The fluorescence of the PI-stained cells was measured at 570 nm using a Cytomics FC 500 flow cytometer (Beckman Coulter Inc., Fullerton, California, USA) and the cell cycle distribution was analyzed using Cytomics FC 500 CXP software.

### 2.5. TUNEL Assay

DNA cleavage was verified by enzymatic end-labeling of DNA strand breaks using in situ cell death detection kits (Roche Applied Science, Indianapolis, IN) according to the manufacturer’s instructions. TUNEL-positive cells were evaluated on a Cytomics FC 500 flow cytometer (Beckman Coulter Inc., Fullerton, California, USA) and the data were analyzed using Cytomics FC 500 CXP software.

### 2.6. Immunoblotting

Whole cell lysates were prepared by sonication for 10 sec in an ice-bath in lysis buffer (1% Triton X-100, 50 mM KCl, 25 mM HEPES, pH 7.8, 10 μg/mL of leupeptin, 20 μg/mL of aprotinin, 125 μM dithiothreitol, 1 mM phenylmethylsulfonyl fluoride, 1 mM sodium orthovanadate). Samples (30 μg of total protein) in 50 μL of reducing sample buffer were boiled for 5 min and resolved on 12.5% SDS polyacrylamide gels for 90 min at 160 volts, then the proteins were transferred onto a polyvinylidene difluoride membrane for 120 min at 60 volts. The membrane was pre-blotted for 1 h at room temperature in blocking buffer (5% skimmed milk in PBS), then incubated for 24 h at 4 °C with primary antibodies against caspase 8, 9, or 3 (all from Cell Signaling Technologies, Danvers, MA, USA), PPARγ, C/EBPα, or β-catenin (all from Santa Cruz Biotechnology, Inc., Santa Cruz, CA, USA), pRb, cyclin D_1_, C/EBPβ, p27, or β-actin (all from Sigma-Aldrich Corp., St. Louis, MO, USA) in blocking buffer, then for 1 h at room temperature with horseradish peroxidase-conjugated secondary antibody (Sigma-Aldrich Corp., St. Louis, MO, USA) in blocking buffer, followed by detection of bound antibody using chemiluminescence reagent (Amersham Biosciences, GE Healthcare, Bucks, UK). To detect multiple signals on a single membrane, the membrane was treated with stripping buffer (59 mM Tris-HCl, 2% SDS, 0.75% 2-mercaptoethanol) for 30 min at 50 °C prior to re-blotting with a different antibody.

### 2.7. Triglyceride Measurement

3T3-L1 adipocytes were homogenized by sonication and triglycerides were measured using commercial kits (DiaSys Diagnostic Systems GmbH & Co. KG, Holzheim, Germany).

### 2.8. Oil Red O Staining 

To examine the lipid accumulation, cells cultured in 12-well plates were fixed with formalin and stained with Oil Red O. To quantify Oil Red O staining, the stained cells were washed with distilled water, and, after removal of the water, 1 mL of isopropanol was added for 10 min and the optical density was measured in a plate reader at 510 nm.

### 2.9. Statistical Analysis

Experiments were repeated four times. The results are expressed as the mean ± SD. Statistical significance was assessed by one way analysis of variance or Student’s t-test, a value of *P* < 0.05 being considered statistically significant.

## 3. Results

### 3.1. Effects of Curcumin on the Viability of 3T3-L1 Preadipocytes and Activation of Caspases

**Figure 1:**
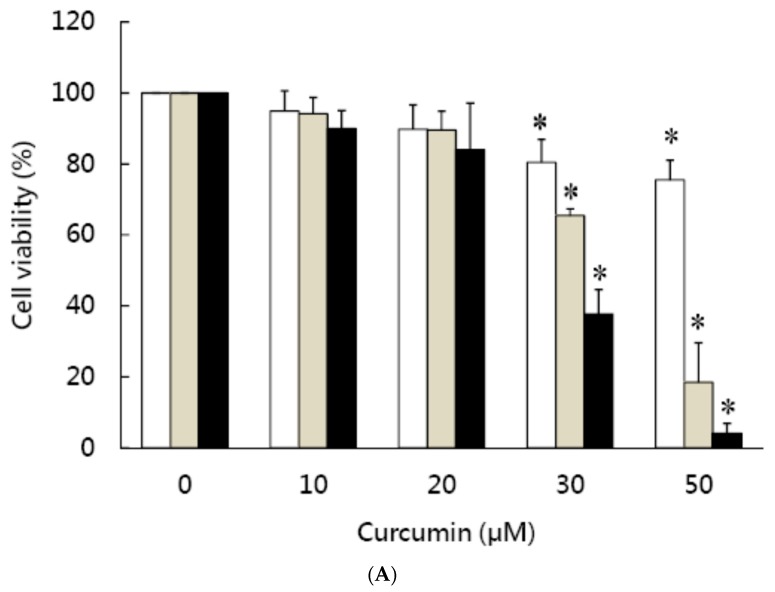

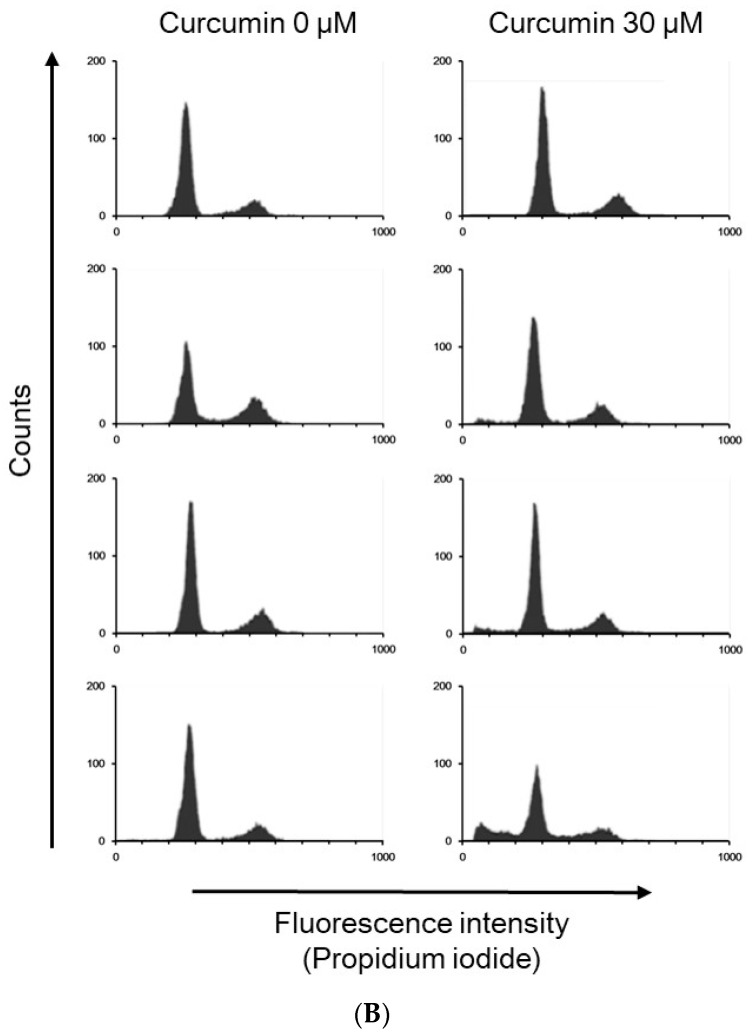
Effect of curcumin on the viability of 3T3-L1 preadipocytes. (**A**) 3T3-L1 preadipocytes were pretreated with 0–50 μM curcumin for 1 h, then were incubated with differentiation medium in the continued presence of the same concentration of curcumin for 24 h (white bars), 48 h (gray bars), or 72 h (black bars) and cell viability was determined using the MTT assay. The results are shown as the mean ± SD for four independent experiments. * *P* < 0.05 compared to the vehicle control. (**B**) 3T3-L1 preadipocytes were pretreated with 0 or 30 μM curcumin for 0–72 h, then the cell cycle distribution was analyzed by flow cytometry. The results shown are representative of those in four separate experiments.

**Figure 2:**
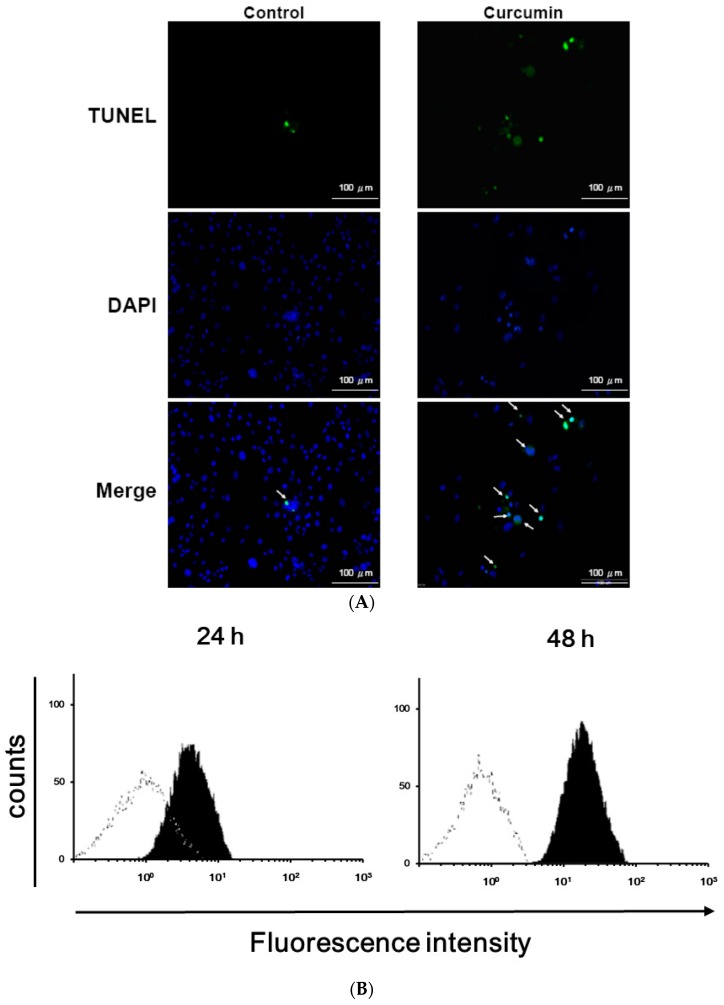

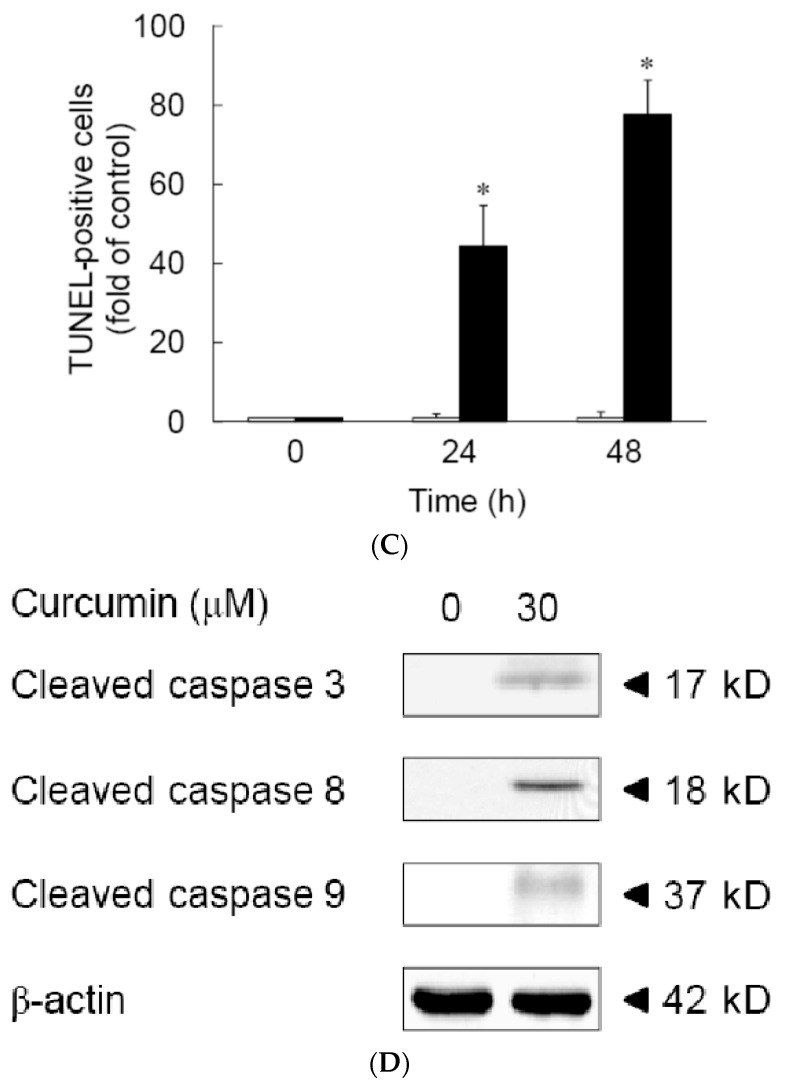
High-dose curcumin induces apoptosis in 3T3-L1 preadipocytes. (**A**–**C**) 3T3-L1 preadipocytes were left untreated (☐) or were incubated with 30 μM curcumin (■) for 24 (**B**–**C**) or 48 h (**B**,**C**), then cell apoptosis was examined by TUNEL staining (blue fluorescence DAPI staining of nuclei and green fluorescence TUNEL staining of apoptotic cells) (**A**) and quantified by flow cytometry (**B**,**C**). (**D**) Western blots of cleaved caspase 3, 8, and 9 in 3T3-L1 preadipocytes after 24-h incubation in medium alone or medium containing 30 μM curcumin. In (**A**,**B**,**D**), the results shown are representative of those in four separate experiments. In (**C**), the results are the mean ± SD for four independent experiments. * *P* < 0.05 compared to the vehicle control.

As shown in Figure 1A, 3T3-L1 preadipocytes were pretreated for 1 h with different concentrations of curcumin (0–50 μM), then incubated with differentiation medium in the continued presence of the same concentration of curcumin for 24 h (white bars), 48 h (gray bars), or 72 h (black bars) and cell viability was determined using the MTT assay, showing that cell survival was not affected by treatment with 10 or 20 μM curcumin, but cell death was induced by 30 or 50 μM curcumin (*P* < 0.05) and the cytotoxic effect was dose- and time-dependent. The LD_50_ of curcumin on preadipocyte viability is about 30 μM. Propidium iodide (PI) staining and flow cytometry analyses showed that 30 μM curcumin caused a time-dependent accumulation of cells with DNA damage in sub-G1 phase (Figure 1B; Table 1). These results showed that high dose curcumin could induce cell death in 3T3-L1 preadipocytes. We then performed a TUNNEL assay to evaluate the apoptotic effect of 30 μM curcumin on 3T3-L1 preadipocytes. As shown in Figure 2A,B, there was a significant increase in high fluorescence intensity cells in the curcumin-treated cells compared to controls after treatment for 24 or 48 h. The quantitative data showed a 40-fold increase at 24 h and a 65-fold increase at 48 h (Figure 2C), demonstrating that this cytotoxic dose of curcumin induced apoptosis in 3T3-L1 preadipocytes.

The activation of caspases is the critical stage in cell apoptosis. To examine the role of caspases in apoptosis induced by curcumin in 3T3-L1 preadipocytes, activation of caspase-8, caspase-9, and caspase-3 was examined after 24 h treatment with 30 μM curcumin. As shown in Figure 2D, curcumin treatment significantly increased the levels of the proteolytically activate forms of caspase-8, caspase-9, and caspase-3. These data suggest that both the intrinsic (caspase-9) and extrinsic (caspase-8) apoptotic pathways are involved in the cytotoxic effects of curcumin. 

### 3.2. Low Dose Curcumin Inhibits Adipogenesis in 3T3-L1 Adipocytes in a Dose-Dependent Manner 

**Figure 3:**
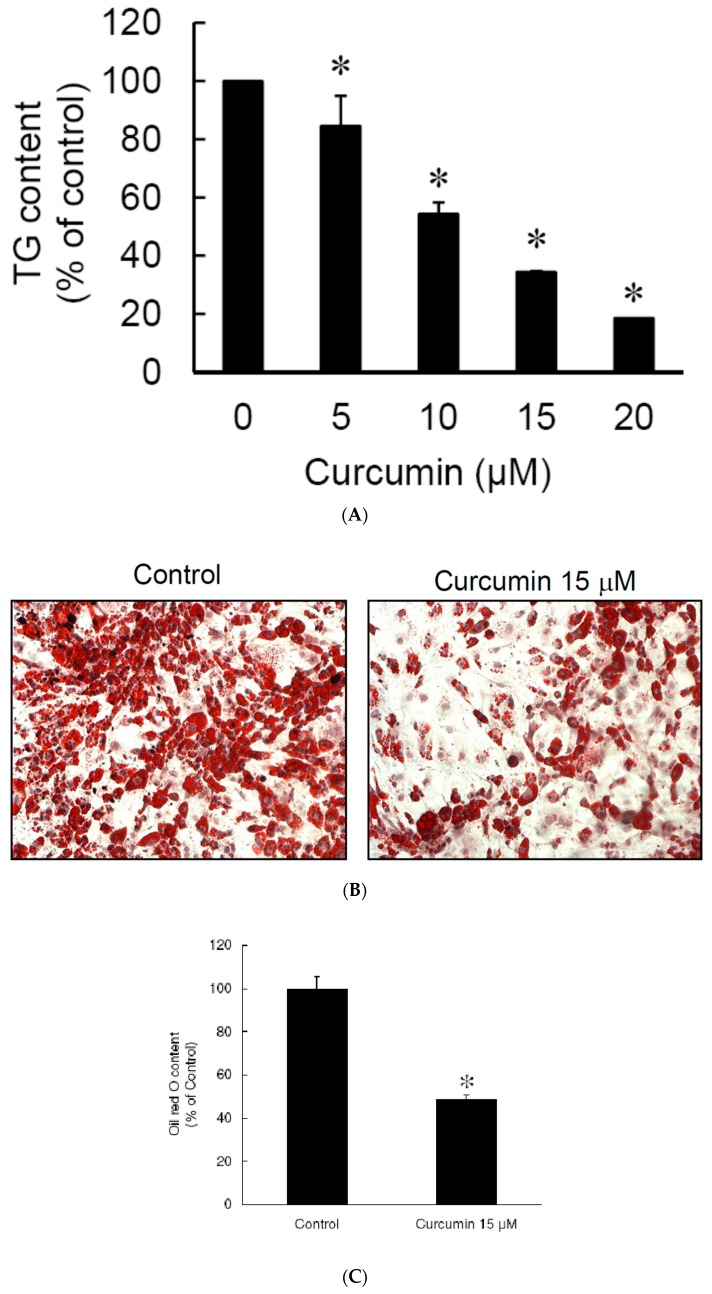
Low dose curcumin inhibits adipogenesis in 3T3-L1 adipocytes. 3T3-L1 preadipocytes were pretreated for 1 h with different concentrations of curcumin (0, 5, 10, 15, 20 μM) (**A**) or with 15 μM curcumin (**B**,**C**), then adipocyte differentiation was induced in the continued presence of the same concentration of curcumin. At the end of differentiation, the intracellular lipid content was determined by measuring triglyceride (TG) (**A**) and Oil red O staining **(B**,**C**). In (**A**,**C**), the results are the mean ± SD for four independent experiments. * *P* < 0.05 compared to the vehicle control. The results shown in (**B**) are representative of those in four separate experiments.

Since the cell viability study showed that low dose (≤20 μM) curcumin did not affect the viability of 3T3-L1 preadipocytes (Figure 1A), we investigated the effect of low dose curcumin on adipocyte differentiation. 3T3-L1 preadipocytes were incubated alone or with 5, 10, 15, or 20 μM curcumin for 1 h, then adipocyte differentiation was induced in the continued presence or absence of the same concentration of curcumin for 9 days, then the intracellular triglyceride content and Oil red O staining were measured to evaluate the efficiency of differentiation. As shown in Figure 3A, curcumin lowered the intracellular triglyceride content in a dose-dependent manner. Furthermore, results of Oil red O staining revealed that 15 μM curcumin significantly inhibited the accumulation of lipid droplets in 3T3-L1 adipocytes (Figure 3B) and this was confirmed by quantitative analyses (Figure 3C). These results show that low dose curcumin has an anti-adipogenic effect on adipocyte differentiation. 

### 3.3. Effect of Low Dose Curcumin on Mitotic Clonal Expansion in 3T3-L1 Cells During the Early Stage of Adipocyte Differentiation

**Figure 4:**
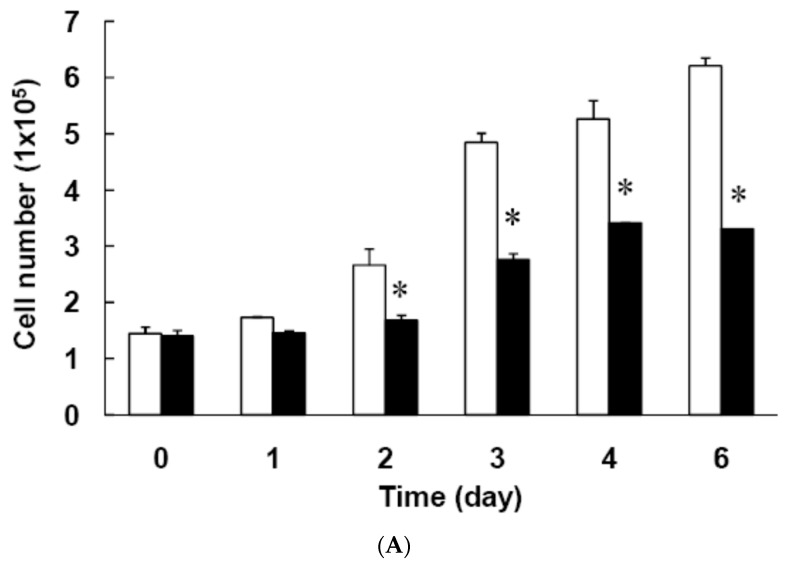

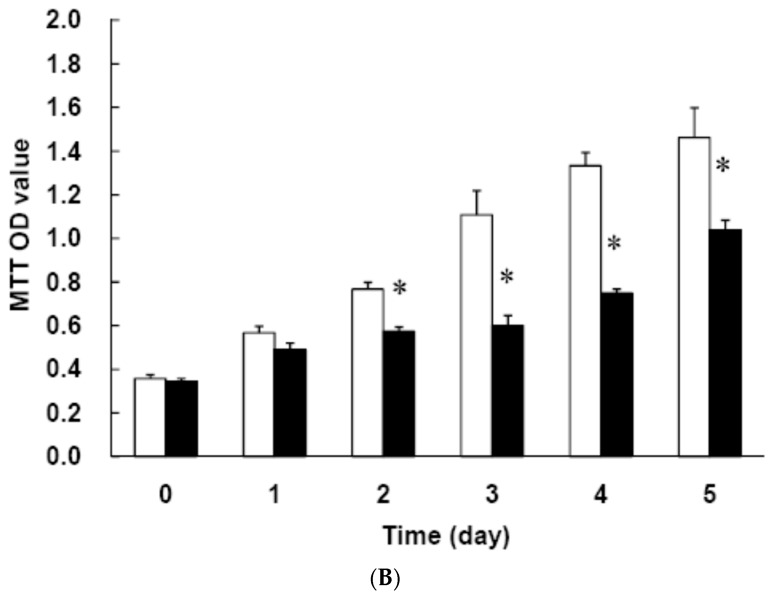
Low dose curcumin inhibits mitotic clonal expansion (MCE) during the early stage of adipocyte differentiation. 3T3-L1 preadipocytes were left untreated (☐) or were pretreated with 15 μM curcumin (■) for 1 h, then adipocyte differentiation was induced in the continued presence or absence of curcumin for 0–5 days, when MCE was measured by cell counting (**A**) or the MTT assay (**B**). The results are the mean ± SD for four independent experiments. * *P* < 0.05 compared to the vehicle control.

**Figure 5:**
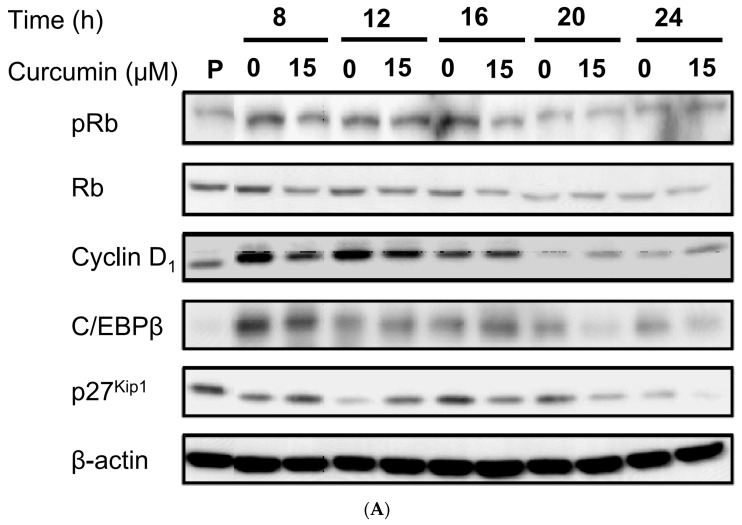

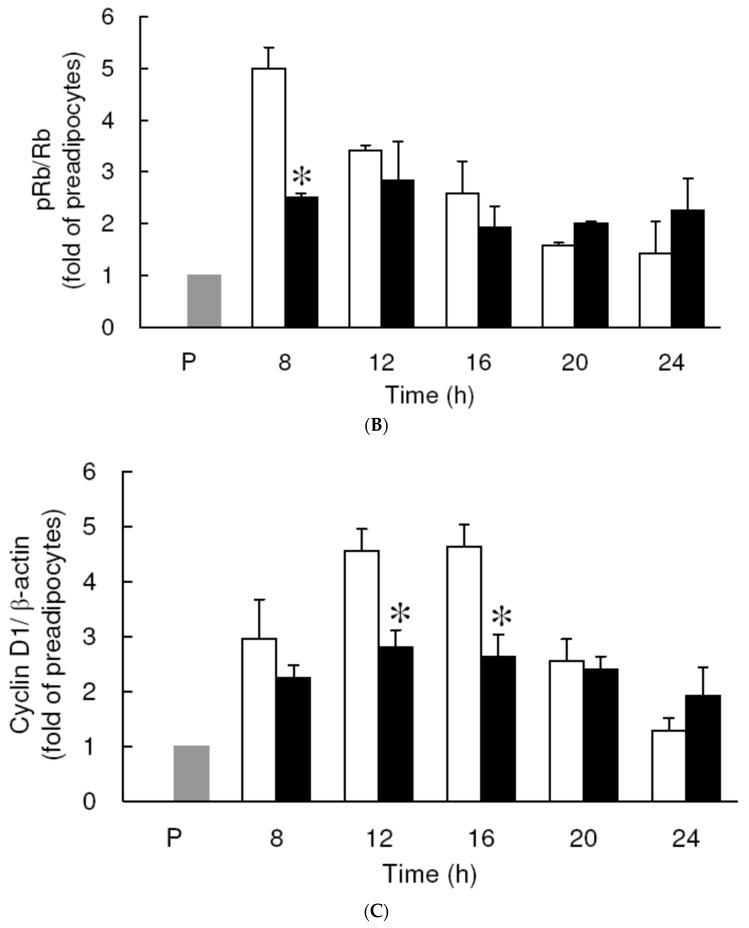

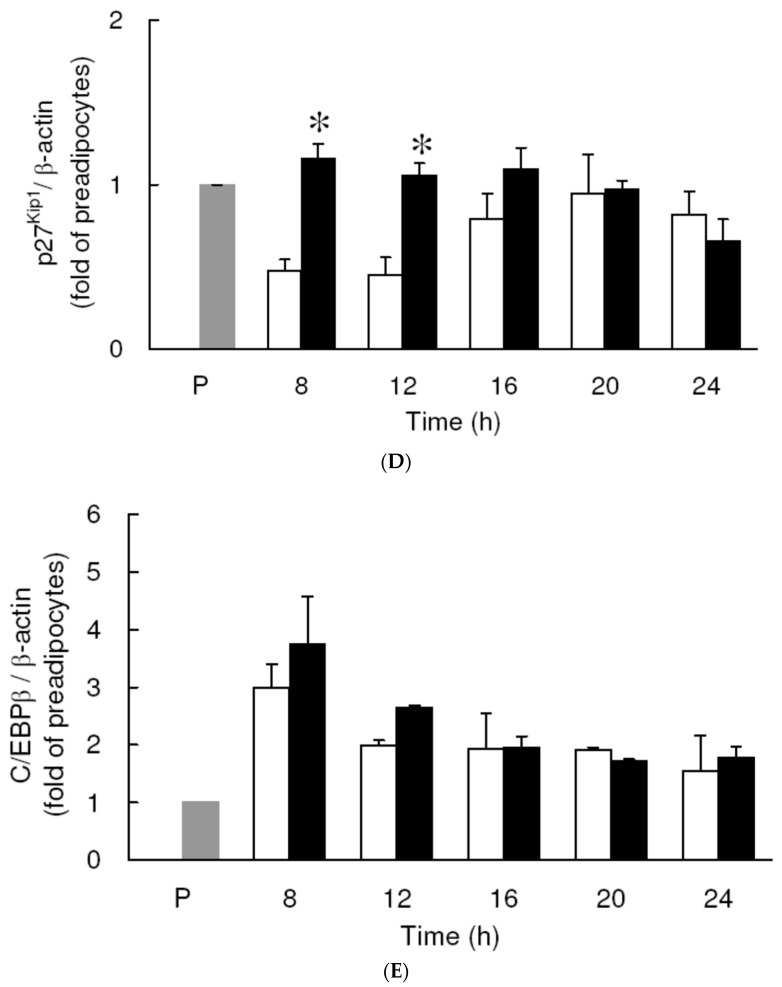
Low dose curcumin alters the expression or phosphorylation of cell cycle regulators during the early stage of adipocyte differentiation. 3T3-L1 preadipocytes were left untreated (☐) or were pretreated with 15 μM curcumin (■) for 1 h, then adipocyte differentiation was induced in the continued presence or absence of curcumin for 8–24 h, when Rb phosphorylation and expression of C/EBPβ, cyclin D_1_, and p27^Kip1^ were measured by immunoblotting. A representative blot is shown in (**A**) and the quantitative analysis of the results in (**B**–**E**). In (A), the results shown are representative of those in four separate experiments. In (**B**–**E**), the results are the mean ± SD for four independent experiments. P, preadipocytes; * *P* < 0.05 compared to the vehicle control at the same time.

First, we explored the effect of 15 μM curcumin on MCE, a necessary step in adipocyte differentiation [19,21,22,23]. MCE of 3T3-L1 preadipocytes following induction of differentiation was found to be significantly decreased by curcumin treatment, as shown by direct cell counting (Figure 4A) or the MTT assay (Figure 4B). Flow cytometry analyses showed that untreated differentiating cells entered the S phase and the subsequent G2/M phase transition after 24 h of induction of adipocyte differentiation and were rearrested in G0/G1 phase of the cell cycle after 48 h of induction of adipocyte differentiation (Figure 1B, Table 2). However, treatment with 15 μM curcumin delayed the S phase entry and the subsequent G2/M phase transition and resulted in an increased number of cells arrested in G0/G1 phase after 24 h of induction of adipocyte differentiation (Figure 1B, Table 2). In addition, curcumin (15 μM)-treated differentiating cells were not rearrested in G0/G1 phase after 72 h of induction of adipocyte differentiation (Table 2). To elucidate the molecular mechanism involved in curcumin-induced delayed cell-cycle entry, we explored the effect of 15 μM curcumin on the cell-cycle regulators by examining phosphorylation of Rb and the expression of cyclin D1, C/EBPβ, and P27^Kip1^. Figure 5A shows a typical result and Figure 5B–E shows the summarized results for each protein. Curcumin treatment significantly decreased Rb phosphorylation in differentiating cells after 8 h of induction of adipocyte differentiation (Figure 5B) and significantly decreased cyclin D1 expression after 12 h and 16 h of induction (Figure 5C). The expression of the Cdk inhibitor P27^Kip1^ was significantly increased in curcumin-treated differentiating cells after 8 h and 12 h of induction of adipocyte differentiation (Figure 5D). There was no difference on expression of C/EBPβ between the two groups (Figure 5E).

### 3.4. Effect of Low Dose Curcumin on Adipogenic Protein Expression in 3T3-L1 Cells During Differentiation

**Figure 6:**
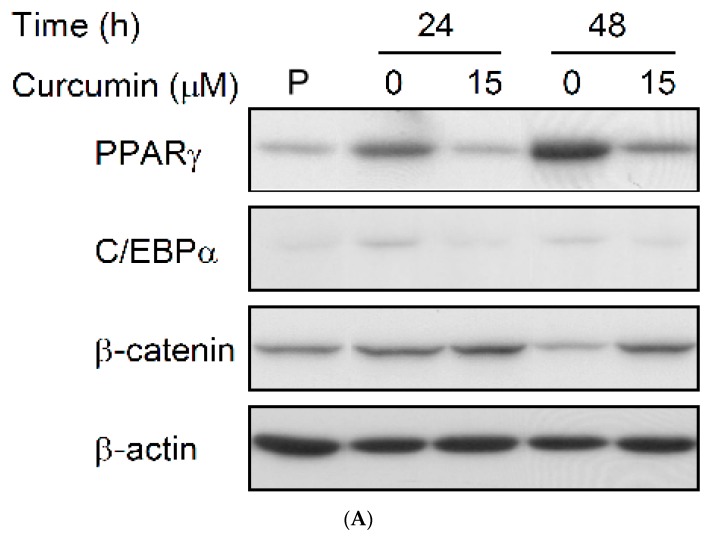

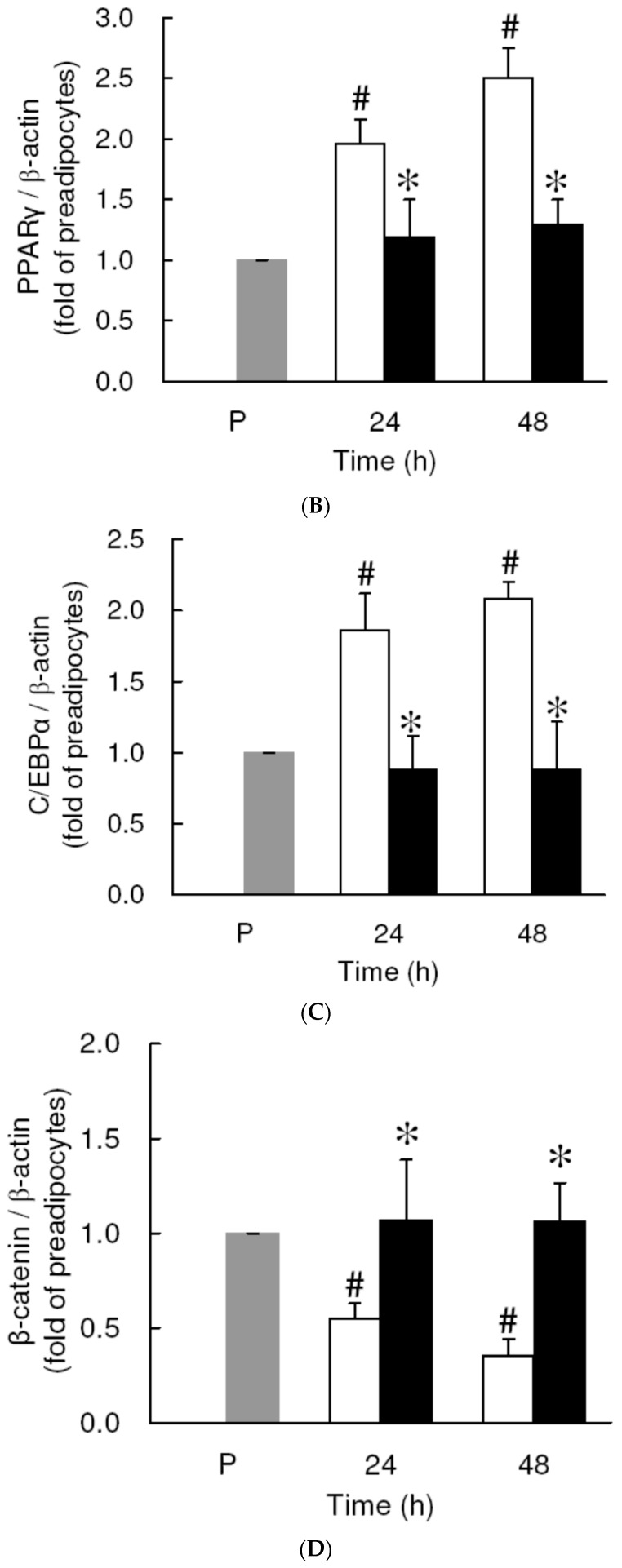
Low dose curcumin alters the expressions of adipogenic proteins during adipocyte differentiation. 3T3-L1 preadipocytes were incubated for 1 h with medium alone (☐) or with 15-μM curcumin (■), then adipocyte differentiation was induced in the continued presence or absence of curcumin for 24 and 48 h and the expression of the adipogenic proteins PPARγ, C/EBPα, and β-catenin was measured by immunoblotting, with β-actin as the loading control. A representative blot is shown in (**A**) and the quantitative analysis in (**B**–**D**). In (A), the results shown are representative of those in four separate experiments. In (**B**–**D**), the results are the mean ± SD for four independent experiments. P, preadipocytes; * *P* < 0.05 compared to the vehicle control at the same time. ^#^ *P* < 0.05 compared to the preadipocytes.

We then explored the effect of low dose curcumin on the expression of the adipogenic transcription factors PPARγ, C/EBPα, and β-catenin. Compared to the levels in preadipocytes, expression of PPARγ (Figure 6A,B) and C/EBPα (Figure 6A,C) in untreated cells was significantly increased after 24 h and 48 h of induction of adipocyte differentiation, while expression of β-catenin was significantly decreased after 48 h of induction (Figure 6A,D). Furthermore, compared to the untreated controls, all three effects were completely or almost completely blocked by 15 μM curcumin. 

### 3.5. Rosiglitazone Cannot Prevent the Curcumin-Induced Inhibition of Adipogenesis in 3T3-L1 Adipocytes

**Figure 7:**
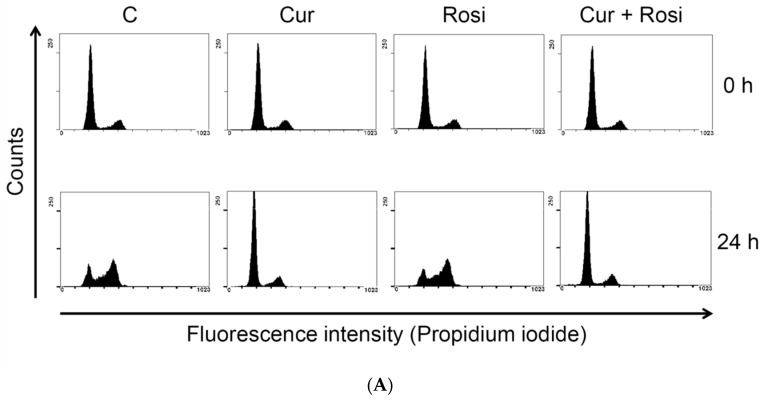

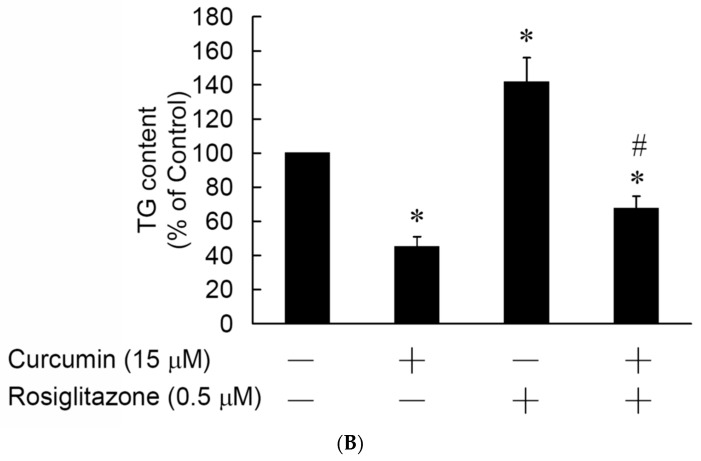
Lack of effect of rosiglitazone on the inhibitory effect of low dose curcumin on MCE and adipocyte differentiation. 3T3-L1 preadipocytes were incubated for 1 h with or without 0.5 µM rosiglitazone, then for 1 h with or without 15 μM curcumin in the continued presence or absence of rosiglitazone, and adipocyte differentiation was induced. During the differentiation processes, changes in MCE were examined at 24 h by flow cytometry (**A**) and changes in the efficiency of adipocyte differentiation were examined at 10 days by TG measurement (**B**). (**A**) is a representative plot of the MCE cytometric analyses from four independent experiments. In (**B**), the bar represents the mean ± SD for four independent experiments. * *P* < 0.05 compared to the vehicle control. ^#^ *P* < 0.05 compared to the rosiglitazone alone.

We then explored whether administration of the PPARγ agonist rosiglitazone inhibited curcumin-inhibited adipogenesis. 3T3-L1 preadipocytes were incubated for 1 h with or without 0.5 μM rosiglitazone, then were incubated for 1 h with or without 15 μM curcumin in the continued presence or absence of rosiglitazone, and changes in MCE and efficiency of adipocytes differentiation was determined, respectively, by flow cytometry and measurement of the intracellular TG content. As shown in Figure 7A, rosiglitazone pretreatment failed to prevent curcumin-induced delayed G1/S phase entry and the subsequent G2/M phase transition in differentiating cells. However, as shown in Figure 7B, adipocyte lipid accumulation was significantly inhibited by curcumin treatment and significantly increased by rosiglitazone treatment, but rosiglitazone pretreatment only partially blocked curcumin-induced inhibition of adipogenesis.

## 4. Discussion

The main finding of this study is that curcumin induced preadipocyte apoptosis in a time- and dose-dependent manner and that this cytotoxic effect involved the activation of both the intrinsic and extrinsic apoptotic pathways. Several studies have demonstrated that curcumin induces apoptosis in different types of cancer cell [35,36,37,38,39,40]. These studies also demonstrated that curcumin induces cancer cell apoptosis through oxidative stress-, ER stress-, caspase cascade-, and mitochondria-dependent pathways. Our data are compatible with these studies. Besides, a recent study demonstrated that curcumin could induce apoptosis in SW872 human adipocytes [41]. Ferguson et al. observed an increasing apoptotic signaling at concentrations greater than 30 μM [42]. Our data show that high dose curcumin induces apoptosis in 3T3-L1 preadipocytes (Figure 1 and Figure 2). In addition, we found that curcumin activates caspase-8 in 3T3-L1 preadipocytes. Lee et al. and Wang et al. found that curcumin could promote Fas and Fas ligand expression in cancer cells [36,37]. Hence, it is possible that Fas/Fas ligand pathway may play a mechanistic role in curcumin-induced preadipocyte apoptosis. Many studies have demonstrated that curcumin suppresses adipocyte differentiation [13,15,43], but its effect on the viability of preadipocytes was not investigated in these studies. An important finding of this work was that curcumin-induced preadipocyte apoptosis could contribute to the inhibitory effect of curcumin on adipogenesis.

Another finding of the present study was that low dose curcumin delayed MCE during the early stage of adipocyte differentiation (Figure 4). The possible mechanism might involve the curcumin-induced downregulation of the early-response cell-cycle regulators phosphorylated Rb (pRb), cyclin D1, and p27^Kip1^ (Figure 5A,C). Such curcumin-induced interference with cell-cycle regulator levels might cause the delayed S phase entry and subsequent G2/M phase transition (Table 2 and Figure 1B) and lead to delayed MCE. Studies have demonstrated that MCE is necessary for adipocyte differentiation [19,21,22,23] and that the critical event for MCE is the transition from G1 phase to S phase [44]. Thus, delayed S phase entry may cause inhibition of MCE and lead to suppression of adipogenesis in 3T3-L1 adipocytes. Our findings are also in agreement with those of several studies which showed that delayed cell cycle entry of 3T3-L1 preadipocytes during the early stage of differentiation decreases MCE and reduces the differentiation efficiency of adipocytes [45,46,47]. Furthermore, we examined whether curcumin-inhibited adipogenesis was blocked by the addition of the PPARγ agonist rosiglitazone, which has been reported to directly bind and activate PPARγ and stimulate adipocyte differentiation [48,49]. Rosiglitazone partially blocked curcumin-suppressed lipid accumulation in 3T3-L1 adipocytes (Figure 7A), but had no effect on curcumin-induced delayed progression of MCE (Figure 7B). These results provide indirect support for the idea that impairment of MCE might lead to the inhibition of adipocyte differentiation. 

Cell-cycle regulators, such as Rb, cyclin D1, and p27^Kip1^, play important roles in the G1 to S phase transition. In quiescent cells, there are low levels of cyclin D1 and high levels of p27^Kip1^, which associates with the cyclin E-Cdk2 complex and suppresses Cdk2 enzyme activity. Mitogenic stimulation (early G1 phase) induces the synthesis of cyclin D1, resulting in increased formation of the cyclin D/Cdk4 complex, then p27^Kip1^ dissociates from the cyclin E-Cdk2 complex and binds to the cyclin D/Cdk4 complex and the Cdk2 activity is enhanced. Activated Cdk2 and Cdk4 phosphorylate Rb, pRb dissociates from E2F1, E2F1-dependent genes are expressed, and the cell cycle starts. Activated cyclin E-Cdk2 also phosphorylates p27^Kip1^, triggering its ubiquitination and degradation [50]. In the present study, the pattern of Rb phosphorylation and cyclin D1 and p27^Kip1^ expression in the control cells matched those in control differentiating cells during the early stage of adipocyte differentiation reported previously [51]. However, treatment with 15 μM curcumin significantly decreased Rb phosphorylation and cyclin D1 expression and increased the P27^Kip1^ expression in the differentiating cells. Tian et al. also reported that treatment of curcumin could induce Rb mRNA upregulation in 3T3-L1 adipocytes [52]. These curcumin-induced changes in the expression profiles of cell cycle regulators might cause delayed G1/S phase transition and lead to the observed reduced MCE and suppressed adipocyte differentiation. Kim et al. [43] reported that curcumin modulates MCE by downregulating cyclin A and Cdk2 levels. During the G1/S phase transition, mitogenic stimulation induces cyclin D1 synthesis, followed sequentially by Rb phosphorylation and Cdk2 activation [50]. In our study, the curcumin-induced decrease in Rb phosphorylation and cyclin D1 expression and increase in P27^Kip1^ expression occurred earlier (G1 phase) than in Kim’s study (S phase), but both studies suggest that curcumin interferes with MCE, then inhibits adipogenesis.

Phosphorylation of Rb is a major step in E2F1 activation, as Rb phosphorylation leads to Rb dissociation from transcription factor E2F1, triggering cell cycle progression [50,53]. Similar events occur during the early stage of adipogenesis [54]. A previous study demonstrated that E2F1 can regulate 3T3-L1 adipogenesis by binding directly to the PPARγ promoter and increasing expression of PPARγ, the master regulator of adipocyte differentiation [51]. In addition, a crosstalk between PPARγ and Rb signaling might operate during adipocyte differentiation, as a study has shown that Rb recruits histone deacetylase 3 (HDAC3) to PPARγ target genes and that disruption of the PPARγ-Rb-HDAC3 complex by Rb phosphorylation or inhibition of HDAC3 activity results in activation of PPARγ, translating as an increase in adipogenesis [55]. In addition, pRb has been shown to interact with the members of the C/EBP family, including C/EBPα and C/EBPβ [16,56]. These findings indicate that pRb plays a positive and direct role in the preadipocyte proliferation and terminal adipocyte differentiation.

In addition to the curcumin-induced downregulation of PPARγ and C/EBPα, we also found that differentiation medium-induced downregulation of β-catenin was prevented by low dose curcumin treatment (Figure 6A,D). Previous studies has demonstrated that upregulation and activation of the Wnt/β-catenin pathway in 3T3-L1 preadipocytes inhibits adipocyte differentiation [32,33]. In the present study, curcumin abolished the differentiation medium-induced downregulation or suppression of Wnt/β-catenin signaling and this may contribute to the curcumin-induced inhibition on adipocyte differentiation. A very similar finding was reported by Ahn et al. [34], who demonstrated that curcumin stimulates the expression of Wnt/β-catenin signaling components and targets in differentiating adipocytes. With the exception of the β-catenin expression results, our study and that of Ahn et al. both show that curcumin might suppress adipogenesis through modulation of Wnt/β-catenin signaling.

**Figure 8:**
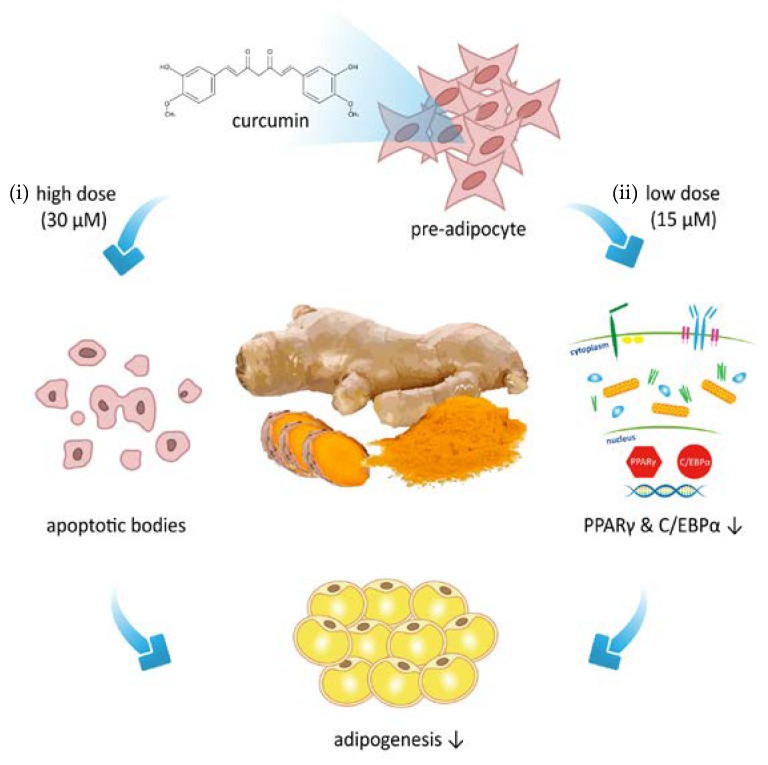
Schematic summary of the regulation of adipogenesis by curcumin in the 3T3-L1 adipocytes. Treatment of 3T3-L1 cells with curcumin has been found to attenuate the adipogenesis through (i) increased preadipocyte apoptosis, and (ii) diminished differentiation signaling protein (PPARγ and C/EBPα) expression. Based on these observations, we identify the dual characteristics of curcumin on the suppression adipogenesis in 3T3-L1 adipocytes.

Results of our present study are compatible with the findings of several studies, including curcumin inhibits adipocyte differentiation through modulation of MCE [43] and activation of Wnt/β-catenin signaling [34,52]. In conclusion, the present study demonstrates that curcumin has dual effects on the regulation of adipogenesis (Figure 8). High dose curcumin induces preadipocyte apoptosis in a time- and dose-dependent manner through caspase 3- 8-, and 9-dependent pathways. In addition, low dose curcumin inhibits adipocyte differentiation by altering the expression of cell cycle regulators, reducing MCE, downregulating expression of PPARγ and C/EBPα, preventing differentiation medium-induced β-catenin downregulation, and decreasing lipid accumulation. These findings suggest that curcumin supplementation could be an effective strategy for treating or preventing development of obesity by a curcumin-induced reduction in the number of preadipocytes and the fat mass of adipocytes.

## Figures and Tables

**Table 1:** Effect of 30 μM curcumin on the cell cycle of 3T3-L1 preadipocytes.

		Cell Cycle Distribution (%)			
		Sub-G1	G0/G1	S	G2/M
0 h	Control	1.28 ± 0.49	75.70 ± 5.08	7.97 ± 2.06	14.56 ± 3.98
	Curcumin	1.09 ± 0.50	74.58 ± 5.62	9.72 ± 4.05	14.20 ± 1.54
24 h	Control	2.47 ± 1.12	47.72 ± 7.28	14.76 ± 2.77	34.24 ± 7.22
	Curcumin	5.33 ± 1.90 *	70.83 ± 4.68 *	11.22 ± 3.98	12.37 ± 1.85 *
48 h	Control	3.88 ± 2.86	71.57 ± 3.96	7.64 ± 1.34	16.08 ± 1.95
	Curcumin	7.36 ± 1.16 *	69.01 ± 7.87	9.83 ± 3.71	13.58 ± 5.19
72 h	Control	4.76 ± 1.86	74.40 ± 3.77	7.56 ± 2.27	12.85 ± 1.81
	Curcumin	18.41 ± 5.65 *	60.83 ± 9.65	10.31 ± 2.94	10.22 ± 1.70

* *P* < 0.05 compared to the control group.

**Table 2:** Effect of 15 μM curcumin on the cell cycle of 3T3-L1 preadipocytes.

		Cell Cycle Distribution (%)		
		G0/G1	S	G2/M
0 h	Control	75.25 ± 3.73	8.37 ± 2.21	15.86 ± 2.16
	Curcumin	75.21 ± 4.87	8.80 ± 2.45	15.57 ± 2.75
24 h	Control	47.10 ± 9.72	15.97 ± 2.58	35.08 ± 9.79
	Curcumin	68.25 ± 4.68 *	16.29 ± 3.98	14.86 ± 3.05 *
48 h	Control	77.52 ± 3.73	7.41 ± 1.21	14.21 ± 1.07
	Curcumin	42.37 ± 9.76 *	13.43 ± 5.16	39.09 ± 9.06 *
72 h	Control	80.26 ± 1.43	7.31 ± 0.62	12.00 ± 0.59
	Curcumin	72.99 ± 2.81 *	8.70 ± 2.44	17.52 ± 3.85 *

* *P* < 0.05 compared to the control group.

## Abbreviations

AMPK	AMP-activated protein kinase
Cdk2	cyclin-dependent kinase 2
C/EBPs	CCAAT enhancer binding proteins
HDAC3	histone deacetylase 3
IBMX	isobutylmethylxanthine
MCE	mitotic clonal expansion
MTT	3-(4,5-dimethylthiazol-2-yl)- 2,5-diphenyltetrazolium bromide
PPARs	peroxisome proliferator-activated receptors
Rb	retinoblastoma protein
TUNEL	terminal deoxynucleotidyl transferase dUTP nick end labeling
